# The impact of health literacy on smoking patterns among male residents: insights from Ningbo City

**DOI:** 10.3389/fpubh.2025.1487400

**Published:** 2025-02-19

**Authors:** Zhenbo Tao, Qianqian Xu, Yingying Zhu, Qiuyan Jin, Lingwei Chen, Shige Ding, Shuning Zhao, Ying Dong

**Affiliations:** Ningbo Municipal Center for Disease Control and Prevention, Ningbo, China

**Keywords:** health literacy, smoking behavior, nonlinear relationship, socio-economic factors, tobacco use

## Abstract

**Background:**

This study examined the associations between health literacy and smoking behaviors among residents in Ningbo City, Zhejiang Province, China, investigating both the impact of health literacy on smoking prevalence and intensity, and its potential role in smoking cessation interventions.

**Methods:**

This cross-sectional study analyzed data from 2,948 male participants in the 2023 Health Literacy and Tobacco Use Surveillance Survey. We applied logistic regression models and restricted cubic spline analyses to assess the association between health literacy and smoking behaviors, adjusting for demographic characteristics, socioeconomic factors, and self-reported health status.

**Results:**

Our findings indicate that higher levels of health literacy are associated with significantly lower rates of smoking (*OR* = 0.643, 95%*CI* = 0.528, 0.783) and daily cigarette consumption (*β* = −1.938, 95%*CI* = −3.649, −0.228). Non-smokers with higher health literacy were more likely to discourage others from smoking (*OR* = 1.464, 95%*CI* = 1.096, 1.955), underscoring health literacy’s crucial role in smoking prevention and control. A nonlinear relationship between health literacy and smoking behavior was identified.

**Conclusion:**

Health literacy significantly influences smoking behavior, with higher literacy levels associated with reduced smoking prevalence and intensity. These findings support incorporating health literacy enhancement into comprehensive smoking cessation strategies.

## Introduction

Tobacco use remains a critical global public health challenge that threatens both population health and social development. According to the World Health Organization (WHO) report in 2019, approximately 1.14 billion people aged 15 years and older use tobacco products globally, demonstrating the substantial scope of this public health crisis (1). This practice precipitates a multitude of diseases and stands as a principal contributor to premature mortality and disability worldwide. The linkage between smoking and an array of health complications, including cardiovascular diseases, cancer, and respiratory disorders, has been thoroughly substantiated through research (2), elevating the health risks associated with tobacco use to a critical public health dilemma (3). The WHO’s findings reveal that tobacco use is responsible for over 8 million deaths annually, with direct smoking attributing to around 7 million fatalities and secondhand smoke exposure causing 1.3 million deaths (1). China, home to nearly 20% of the global population, is responsible for more than 40% of the world’s total cigarette consumption, leading to over 1 million tobacco-related deaths each year, predominantly among male smokers (4). Research has identified smoking as the leading risk factor for male mortality (5), emphasizing the urgent need for comprehensive strategies to address this public health threat.

In the modern era, health literacy (HL) has gained significant traction as a subject of vital concern, referring to an individual’s ability to obtain, understand, evaluate, and apply health-related information to make informed decisions and actions affecting their health (6). Research underscores that insufficient HL can elevate mortality and hospitalization risks in individuals with chronic conditions (7), and adversely affect daily dietary choices (8). Conversely, enhanced HL is linked to improved health outcomes in children and adolescents (9), a decreased propensity for health-risk behaviors, and fewer negative health consequences (10). Moreover, HL plays a crucial role in heightening awareness about pressing health challenges, including COVID-19 (11). As reported in 2022, the average HL level among Chinese residents was a mere 23.15%, revealing that a staggering majority, over three-quarters of the population, possesses inadequate HL skills (12). This stark reality underscores the urgent need for targeted interventions to bolster HL, aiming to improve public health outcomes and resilience against health threats.

Recent studies have illuminated various risk factors that influence smoking behavior, underscoring the complex interplay between HL and such behaviors. Factors affecting HL, including age, socioeconomic status, education, and occupation, have shown significant overlap with determinants of smoking habits (5, 13). This correlation suggests that populations with lower HL levels frequently exhibit higher rates of smoking. Furthermore, evidence indicates a pronounced link between HL and smoking-related behaviors and outcomes; individuals with greater HL are more inclined toward initiating smoking cessation efforts (14) and exhibit a reduced tendency for relapse following cessation interventions (15). However, the relationship between HL, awareness of smoking hazards, the intensity of smoking habits, and the discouragement of smoking among peers remains underexplored.

This study aims to address these knowledge gaps by examining the relationship between HL and smoking behaviors among residents of Ningbo City, with three specific objectives: (1) to examine the association between HL and smoking patterns, (2) to assess the influence of HL on smoking intensity, and (3) to evaluate HL’s role in promoting smoking prevention through social networks. These findings could inform the development of smoking cessation interventions that integrate HL as a key component of tobacco control strategies.

## Methods

### Study population

This manuscript describes a cross-sectional investigation leveraging data from the 2023 Health Literacy and Tobacco Use Surveillance Survey, executed among the populace of Ningbo City, Zhejiang Province. The cohort for this study comprised permanent residents within the age bracket of 15 to 69 years, who had been residing continuously in the designated survey area for a minimum duration of 6 months.

Prior to initiating the survey, comprehensive details concerning the study’s objectives, alongside the voluntary and anonymous nature of participation, were communicated to all prospective respondents. Participants were required to have a thorough understanding of the provided written informed consent information and to have explicitly agreed to partake in the survey beforehand.

### Sampling methods

The sampling framework of this investigation was anchored in the broader context of the Health Literacy and Tobacco Use Surveillance in Zhejiang Province. The primary aim was to elucidate the prevalence of HL among the provincial residents. To this end, a meticulous sample size determination was employed, guided by the following methodology:

The minimal requisite sample size for each county (district) was ascertained using the formula *N* = $\frac{{\mu_{\alpha }}^{2}*p*\left(1-p\right)}{\delta^{2}}$ *deff. In 2019, the level of HL in Zhejiang was 29.49%, p = 0.2949, the allowable relative error was set to 15%, and the allowable absolute error δ = 0.2949 * 0.15 = 0.0442, $\mu_{\alpha }$ = 1.96, deff = 1. This calculation suggested a base sample size of 408 for each layer. Considering potential non-responses and invalid questionnaires, the sample size was adjusted to 640 for each county (district). Given Ningbo’s administrative structure comprising 10 counties (districts), the aggregate initial sample size was established at 6400 participants. Notably, the analysis was confined to male smokers due to the negligible representation of female smokers in the sample, with only nine instances reported. Following the exclusion of female participants and invalid questionnaires, the refined sample consisted of 2,948 respondents.

A stratified multi-stage probability sampling method proportional to population size was used in this study. The complete sampling process involved four sequential stages: (1) Ten counties (districts) in Ningbo City were each represented by the selection of four townships. (2) Within each chosen township, two segments were identified for further sampling. (3) A random selection of 120 households was conducted within each segment. (4) Utilizing a Kish grid, one participant was chosen from each household. This structured sampling approach was designed to ensure a representative and statistically robust sample, facilitating an accurate assessment of HL and tobacco use patterns within the targeted population.

### Tools used

The survey was meticulously conducted utilizing the Health Literacy and Tobacco Use Surveillance questionnaire, a comprehensive instrument partitioned into three distinct sections: personal characteristics, HL and smoking status, officially sanctioned by the Chinese Health Education Center. The reliability of the questionnaire was validated through an overall Cronbach’s alpha and Spearman-Brown coefficient of 0.95 and 0.94, respectively (16), indicating high internal consistency and reliability.

The first section collected demographic and personal information, including sex, age, marital status, educational attainment, profession, annual household income, and self-rated health status. These variables were essential for examining the factors influencing health literacy and smoking behaviors in the study population.

The subsequent section delved into HL, incorporating a variety of question types to assess this multifaceted concept. The HL assessment instrument comprises six core dimensions: scientific perspectives on health, prevention of infectious diseases, prevention of non-communicable diseases, safety and emergency response, basic healthcare services, and health information literacy. The questions were categorized into three formats: true/false (awarding 1 point for each correct response), single-answer multiple choice (with only one correct option, also yielding 1 point per correct answer), and multiple-answer multiple choice (where a correct response necessitated identifying all correct options without any incorrect selections). The aggregate maximum score attainable on this scale was 66 points, with a threshold score of 53 (representing 80% of the total score) delineated as the benchmark for adequate HL. Conversely, scores ranging from 0 to 52 were indicative of insufficient HL levels. The proportion of participants demonstrating sufficient HL relative to the total participant count was utilized to define the HL level (17).

The final part of the questionnaire concentrated on smoking behaviors, including the prevalence and frequency of smoking, alongside awareness regarding the hazards associated with smoking and second-hand smoke exposure. The assessment of knowledge concerning smoking dangers was structured around seven single-answer questions, with a full comprehension of the correct responses equated to an awareness of smoking-related risks. The specifics of these questions and their respective options are detailed in Table 1.

**Table 1:** Variable detail definition.

Variable	Definition
Health literacy	limited = score < 53; adequate = score > = 53
Knowledge of smoking hazards	limited = score < 7; adequate = score > = 7
Smoking can cause stroke	0 = no/not sure; 1 = yes
Smoking can cause heart disease	
Smoking can cause lung cancer	
Smoking can cause erectile dysfunction	
Secondhand smoke can cause heart disease in adults	
Secondhand smoke can cause lung disease in children	
Secondhand smoke can cause lung cancer in adults	

The survey was administered by trained investigators who completed standardized training before data collection to ensure consistency and data quality across all survey activities.

### Statistical analysis

Data analysis was conducted using SPSS version 26.0 and R version 4.3.1. Statistical significance was set at a two-tailed *p* < 0.05. The *Kolmogorov–Smirnov* test assessed variable normality. For non-normally distributed variables, median values with quartiles were reported and compared using the *Mann–Whitney U* test. Categorical and ordinal variables were presented as ratios and compared using *Pearson chi-square* and *Mann–Whitney U* tests. Multiple logistic regression models were developed to examine relationships between smoking behavior, HL, and other variables, adjusting for covariates. A forest plot visualized the association between smoking habits and HL.

The cohort was bifurcated based on smoking status. Among smokers, a linear regression model was constructed to investigate the correlation between the frequency of weekly smoking and HL. Conversely, for non-smokers, a logistic regression model was devised to examine the linkage between the propensity to discourage smoking and HL. Within each model, key statistics including odds ratios (*OR*) or regression coefficients (*β*), 95% confidence intervals (*CI*), and *p*-values were systematically reported to elucidate the strength and significance of observed associations.

Lastly, to probe potential non-linear dynamics between smoking behaviors and HL, a restricted cubic spline (RCS) analysis was conducted. This sophisticated statistical technique allows for the exploration of complex relationships that might not be readily apparent or adequately described by linear models, offering nuanced insights into how varying levels of HL might influence smoking habits.

## Results

### Basic characteristics

Definitions of HL and tobacco harm knowledge are presented in Table 1. Among 2,948 participants, 1,149 (38.98%) were smokers (Table 2). The prevalence of adequate HL was significantly lower in smokers (25.9%) compared to non-smokers (40.2%, *p* < 0.05).

**Table 2:** Association between smoking status and basic characteristics.

Variable	Non-smoking group (*n* = 1799)	Smoking group (*n* = 1,149)	Z/χ^2^	*p*
Health literacy			63.385	<0.001
Limited	1,075(59.8)	851(74.1)		
Adequate	724(40.2)	298(25.9)		
Age(years)	49.00(37.00,60.00)	53.00(42.00,60.00)	−5.799	<0.001
Education levels			−7.975	<0.001
Illiterate or elementary school	298(16.6)	253(22.0)		
Junior high school	526(29.2)	430(37.4)		
High school	393(21.8)	249(21.7)		
College or above	582(32.4)	217(18.9)		
Occupation			30.245	<0.001
Public sectors	219(12.2)	111(9.7)		
Office, student, or other non-manual	648(36.0)	336(29.2)		
Agriculture	358(19.9)	262(22.8)		
Factory or manual	386(21.5)	265(23.1)		
Other	188(10.5)	175(15.2)		
Region			0.189	0.664
Rural	752(41.8)	471(41.0)		
Urban	1,047(58.2)	678(59.0)		
Marital status			54.510	<0.001
Single/Widow/Divorced	272(15.1)	71(6.2)		
Married	1,527(84.9)	1,078(93.8)		
Annual household income			0.346	0.557
≤100,000	1,141(63.4)	741(64.5)		
>100,000	658(36.6)	408(35.5)		
Self-reported good health			12.464	<0.001
Yes	1,245(69.2)	723(62.9)		
No	554(30.8)	426(37.1)		
Knowledge of smoking hazards			14.284	<0.001
Limited	1,088(60.5)	774(67.4)		
Adequate	711(39.5)	375(32.6)		
Conditions smoking causes				
Stroke			38.230	<0.001
No	582(29.3)	464(40.4)		
Yes	1,271(70.7)	685(59.6)		
Heart attack			21.414	<0.001
No	621(34.5)	494(43.0)		
Yes	1,178(65.5)	655(57.0)		
Lung cancer			30.970	<0.001
No	95(5.3)	124(10.8)		
Yes	1704(94.7)	1,025(89.2)		
Erectile dysfunction			17.821	<0.001
No	887(49.3)	658(57.3)		
Yes	912(50.7)	491(42.7)		
Conditions secondhand smoke causes			18.355	<0.001
Heart diseases in adults	544(30.2)	435(37.9)		
No	1,255(69.8)	714(62.1)		
Yes				
Lung illnesses in children			20.933	<0.001
No	142(7.9)	150(13.1)		
Yes	1,657(92.1)	999(86.9)		
Lung cancer in adults			42.445	<0.001
No	124(6.9)	163(14.2)		
Yes	1,675(93.1)	986(85.8)		
Exposure to secondhand smoke			289.841	<0.001
No	825(45.9)	177(15.4)		
Yes	974(54.1)	972(84.6)		

Significant differences between smokers and non-smokers were observed in demographic and socio-economic characteristics. Smokers were generally older and had lower educational attainment. Smoking rates were lower among public sector employees, office workers, and students, but higher among agricultural and manual laborers. A higher proportion of smokers were married, and fewer smokers reported good self-rated health compared to non-smokers (*p* < 0.05 for all comparisons).

Annual income and urban–rural distribution showed no significant differences between smokers and non-smokers (*p* > 0.05). However, non-smokers demonstrated better awareness of tobacco-related health risks, including both direct smoking and secondhand smoke exposure (*p* < 0.05).

### Analysis of factors influencing smoking behavior

**Figure 1:**
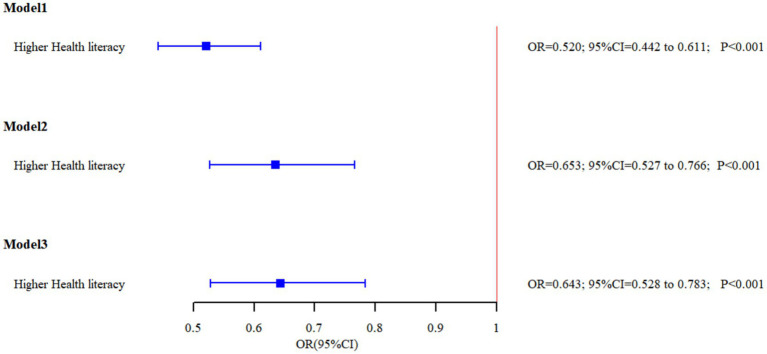
The relationship between HL and smoking behavior in the three models.

Logistic regression analyses of smoking determinants are presented in Table 3 and Figure 1. Three sequential models were constructed: Model 1 (unadjusted), Model 2 (adjusted for sociodemographic factors including age, education level, occupation, urban–rural status, marital status, annual income, and self-rated health), and Model 3 (further adjusted for smoking harm awareness and secondhand smoke exposure).

**Table 3:** Logistic regression analysis of smoking behavior.

Variable	M1		M2		M3	
	*p*	OR(95%CI)	*p*	OR(95%CI)	*p*	OR(95%CI)
Adequate HL	<0.001	0.520(0.442, 0.611)	<0.001	0.635(0.527, 0.766)	<0.001	0.643(0.528, 0.783)
Age			0.737	0.999(0.99, 1.007)	0.656	1.002(0.993, 1.011)
Education levels			<0.001	0.799(0.716, 0.892)	<0.001	0.794(0.707, 0.89)
Occupation (Ref = Public sectors)						
Office, student, or other non-manual			0.618	0.932(0.706, 1.23)	0.413	0.886(0.662, 1.184)
Agriculture			0.316	0.842(0.602, 1.178)	0.084	0.733(0.516, 1.043)
Factory or manual			0.518	0.903(0.663, 1.231)	0.167	0.795(0.575, 1.1)
Other			0.208	1.237(0.888, 1.724)	0.613	1.094(0.772, 1.55)
Region (Urban)			0.233	0.907(0.772, 1.065)	0.392	0.929(0.784, 1.1)
Marital status (Married)			<0.001	2.237(1.635, 3.06)	<0.001	1.81(1.307, 2.505)
Annual household income (>100,000)			0.008	1.271(1.066, 1.517)	0.020	1.247(1.036, 1.501)
Self-reported good health (no)			0.030	1.198(1.018, 1.409)	0.196	1.119(0.944, 1.327)
Knowledge of smoking hazards (Adequate)					0.103	0.867(0.731, 1.029)
Exposure to secondhand smoke (Yes)					<0.001	4.515(3.735, 5.457)

Across the three models, possessing HL consistently emerges as a protective factor against smoking, with individuals possessing HL showing lower rates of smoking (*OR* < 1). In Model 3, individuals with adequate HL exhibited a 35.7% reduction in smoking risk (*OR* = 0.643, 95%*CI* = 0.528, 0.783). Higher education levels are associated with lower smoking rates. Married individuals show higher smoking rates. Exposure to secondhand smoke on a regular basis is associated with higher smoking rates (*OR* = 4.515, 95%*CI* = 3.735, 5.457), indicating that individuals regularly exposed to secondhand smoke are 4.5 times more likely to be smokers compared to those without such exposure. However, awareness of the harms of smoking did not show a statistically significant impact on smoking behavior.

### Analysis of influencing factors of smoking severity

Table 4 presents the linear regression analysis of daily cigarette consumption among smokers. Adequate HL was consistently associated with lower smoking intensity across all models (*β* < 0). In the fully adjusted Model 3, adequate HL was associated with smoking approximately two fewer cigarettes per day (*β* = −1.938, 95%*CI* = −3.649, −0.228). Age showed a positive association with daily cigarette consumption, while higher education levels were associated with smoking fewer cigarettes. No other variables showed significant associations in the final model.

**Table 4:** Linear regression analysis of smoking severity.

Variable	M1		M2		M3	
	*p*	β(95%CI)	*p*	β(95%CI)	*p*	β(95%CI)
Adequate HL	<0.001	−4.661(−6.204, −3.118)	0.032	−1.858(−3.555, −0.160)	0.026	−1.938(−3.649, −0.228)
Age			<0.001	0.168(0.092, 0.245)	<0.001	0.172(0.095, 0.249)
Education levels			0.025	−1.041(−1.948, −0.133)	0.021	−1.076(−1.986, −0.165)
Occupation (Ref = Public sectors)						
Office, student, or other non-manual			0.918	−0.132(−2.649, 2.385)	0.936	−0.103(−2.627, 2.421)
Agriculture			0.225	1.818(−1.12, 4.756)	0.208	1.900(−1.060, 4.860)
Factory or manual			0.984	−0.028(−2.772, 2.716)	0.980	0.035(−2.72, 2.791)
Other			0.278	1.572(−1.268, 4.411)	0.255	1.657(−1.199, 4.512)
Region (Urban)			0.369	0.645(−0.763, 2.052)	0.323	0.712(−0.701, 2.125)
Marital status (Married)			0.493	1.011(−1.878, 3.900)	0.547	0.890(−2.01, 3.789)
Annual household income (>100,000)			0.168	1.060(−0.449, 2.568)	0.150	1.113(−0.402, 2.628)
Self-reported good health (no)			0.134	−1.056(−2.44, 0.327)	0.121	−1.096(−2.482, 0.290)
Knowledge of smoking hazards (Adequate)					0.415	0.605(−0.849, 2.058)
Exposure to secondhand smoke (Yes)					0.430	0.748(−1.109, 2.604)

### Analysis of influencing factors to dissuade others from smoking

The factors influencing non-smokers’ likelihood to discourage others from smoking were examined using logistic regression (Table 5). Adequate HL was consistently associated with a greater likelihood of discouraging smoking across all models. In the fully adjusted Model 3, individuals with adequate HL were 46.4% more likely to discourage others from smoking (*OR* = 1.464, 95%*CI* = 1.096, 1.955). Conversely, individuals who encounter secondhand smoke in their daily lives were less inclined to discourage smoking in others (*OR* = 0.527, 95%*CI* = 0.409, 0.680), indicating a potential desensitization or resignation to the prevalence of smoking behaviors. Other variables, such as age, educational level, and occupation, did not demonstrate statistical significance in Model 3.

**Table 5:** Logistic regression analysis of dissuading others from smoking.

Variable	M1		M2		M3	
	*p*	OR(95%CI)	*p*	OR(95%CI)	*p*	OR(95%CI)
Adequate HL	0.012	1.375(1.071, 1.766)	0.018	1.411(1.061, 1.875)	0.010	1.464(1.096, 1.955)
Age			0.990	1(0.987, 1.014)	0.892	0.999(0.986, 1.013)
Education levels			0.334	0.917(0.77, 1.093)	0.325	0.916(0.768, 1.091)
Occupation (Ref = Public sectors)						
Office, student, or other non-manual			0.111	1.388(0.928, 2.078)	0.087	1.426(0.950, 2.141)
Agriculture			0.556	0.859(0.517, 1.426)	0.725	0.912(0.548, 1.521)
Factory or manual			0.258	1.310(0.821, 2.090)	0.155	1.407(0.878, 2.255)
Other			0.943	0.981(0.588, 1.639)	0.889	1.037(0.619, 1.739)
Region (Urban)			0.248	1.162(0.901, 1.499)	0.297	1.146(0.887, 1.481)
Marital status (Married)			0.229	0.761(0.487, 1.188)	0.429	0.835(0.534, 1.306)
Annual household income (>100,000)			0.534	0.915(0.692, 1.210)	0.570	0.922(0.695, 1.222)
Self-reported good health (no)			0.514	0.916(0.704, 1.192)	0.656	0.941(0.721, 1.229)
Knowledge of smoking hazards (Adequate)					0.269	0.867(0.673, 1.117)
Exposure to secondhand smoke (Yes)					<0.001	0.527(0.409, 0.680)

### Nonlinear analysis of health literacy and smoking

**Figure 2:**
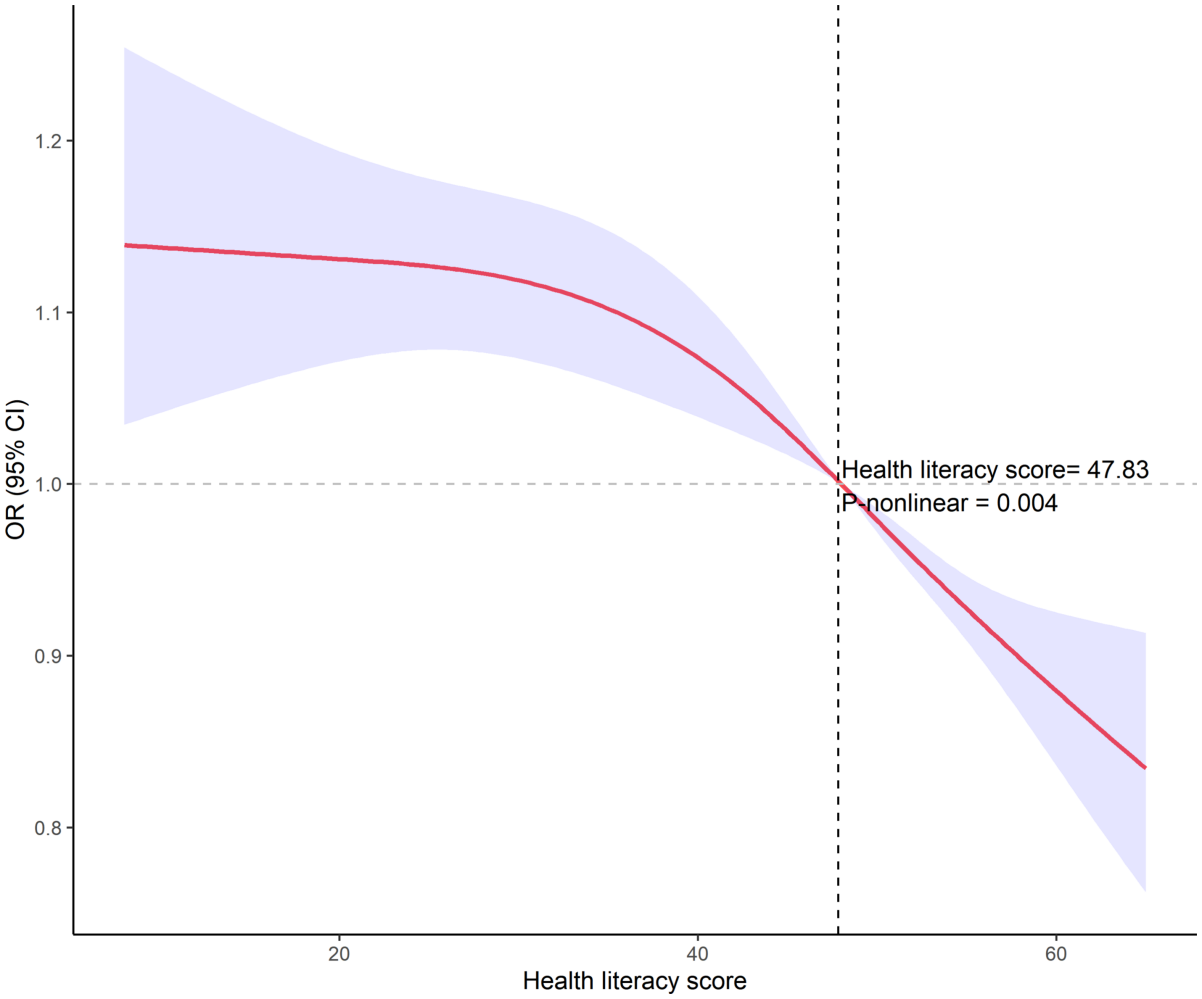
RCS analysis of health literacy and smoking behavior.

As shown in Figure 2, utilizing restricted cubic spline analysis, a nonlinear relationship between HL and smoking behavior was identified (*p* = 0.004). The inflection point was determined to be at a score of 47.83, it shows that the when HL is raised to this threshold, smoking behavior changes more.

## Discussion

### The relationship between smoking behavior and health literacy

This study, conducted among the permanent residents of Ningbo City, examines the relationship between HL and smoking behavior, including the severity of such habits. Analyzing data from 2,948 participants, the study results in Table 2 showed that individuals with higher levels of HL exhibit significantly lower smoking rates compared to those with lower HL. After covariate-adjusted multifactor logistic regression, the results are still robust (Table 3; Figure 1). This observation aligns with previous research outcomes (12). HL levels influence both smoking awareness and behavior. Individuals with higher HL demonstrate better understanding of smoking’s health risks and greater tendency toward health-promoting behaviors, including smoking cessation and avoidance of smoking environments. In contrast, those with lower HL may have limited understanding of smoking risks, increasing their vulnerability to smoking culture influences. These findings emphasize the importance of enhancing population-level HL for effective smoking prevention and control.

Further analysis revealed additional dimensions of HL’s influence on smoking behavior. Among smokers, higher HL was associated with lower daily cigarette consumption (Table 4), demonstrating that HL affects not only smoking initiation but also smoking intensity. Moreover, non-smokers with higher HL levels showed greater likelihood of discouraging others from smoking (Table 5), suggesting HL’s role in promoting smoking prevention through social networks. The restricted cubic spline analysis revealed a significant nonlinear relationship between HL and smoking behavior, with an inflection point at a score of 47.83 (Figure 2). The analysis demonstrated that the protective effect against smoking was most pronounced at HL scores above this threshold, with progressively decreasing odds of smoking as HL levels increased. This nonlinear relationship provides valuable insights for public health interventions, suggesting that efforts to improve population HL levels could yield substantial benefits for smoking control.

### The relationship between smoking behavior, age, and education level

Beyond the relationship between HL and smoking, our analysis revealed significant demographic differences between smokers and non-smokers, particularly regarding age and education. Higher smoking rates among older individuals suggest generational variations in health behavior perceptions and responses to different life stage pressures. In alignment with the bulk of existing research, individuals with higher educational attainment exhibit a lower risk of smoking, a risk that escalates with age (18, 19). Those with a higher level of education have easier access to information about the harms of tobacco and are more likely to engage in and practice smoking cessation behaviors, demonstrating a propensity toward healthier lifestyles (20). Conversely, individuals with lower educational levels may exhibit a weaker response to health promotion, possess less knowledge about the health risks associated with smoking, and have fewer opportunities to access smoking cessation services. This scenario potentially leads to a higher incidence of smoking and, subsequently, a greater dependency on tobacco (21).

### The relationship between smoking behavior and cognition

Our study revealed important connections between health awareness, risk perception, and smoking behavior. Participants who demonstrated better understanding of smoking risks and reported positive self-assessed health status were less likely to smoke, highlighting the crucial role of health cognition in behavioral choices.

However, significant misconceptions about smoking risks persist in the population. For example, only 55.61% of current smokers correctly identified that low-tar cigarettes are not less harmful than regular cigarettes. This finding suggests that tobacco industry marketing strategies continue to influence public understanding of smoking risks (4).

The relationship between health cognition and smoking behavior appears to operate through multiple pathways. Individuals with positive health self-assessment tend to adopt healthier lifestyles overall, including avoiding smoking. This may reflect both heightened health consciousness and sociopsychological factors, as these individuals often maintain their healthy behaviors to align with their self-image and societal health standards.

These findings emphasize the importance of comprehensive health education in smoking prevention. Strengthening public understanding of smoking risks, particularly regarding chronic disease associations, could effectively reduce smoking rates. Moreover, improving health literacy through both educational and economic interventions may enhance individuals’ capacity to access, understand, and act upon health information, thereby promoting healthier lifestyle choices.

### Family and work environment influences on smoking behavior

The link between marital status and smoking behavior may highlight the relationship between social support and individual choices regarding behavior. The increased smoking rates among married individuals could relate to life stress, a spouse’s smoking habits, or broader socio-cultural factors (22). Those who are cohabiting or married tend to bear more societal and familial responsibilities, leading to a higher risk of smoking. This observation underscores the potential efficacy of family and community-level interventions, such as enhancing support for healthy lifestyles within families and encouraging partners to quit smoking together.

Occupationally, the prevalence of smoking is comparatively lower among those working in the public sector, office environments, students, or other non-manual labor roles, while individuals engaged in agriculture, factory work, or manual labor have significantly higher smoking rates. Several factors could drive this disparity. Firstly, occupation types often correlate with an individual’s education level and socio-economic status. Typically, higher education and socio-economic status are associated with healthier lifestyle choices, likely due to easier access to health-related information, including the risks of smoking, thus reducing the occurrence of smoking behaviors. Secondly, the work environment itself may directly impact smoking habits. For example, agriculture and manual labor are often associated with higher job stress and physical fatigue, where individuals might use smoking as a stress relief method. Moreover, these sectors might lack health promotion policies and smoking control measures, such as less stringent enforcement of workplace smoking bans compared to office settings.

Our findings reveal that individuals exposed to secondhand smoke in their daily lives are at a high risk of current smoking (*OR* = 4.515, 95%*CI* = 3.735, 5.457). Exposure to secondhand smoke could lead to smoking due to imitation or social needs (23). Both domestic environments and public spaces should be key venues for promoting smoke-free and smoking control initiatives. Particularly, adolescents, susceptible to external influences, may easily mimic others’ smoking behaviors (24). It is recommended to establish smoke-free homes on top of banning smoking in public places to reduce youth exposure to tobacco and lower the overall smoking rates in the population (25).

### Limitations

This study also has some limitations. Due to its cross-sectional design, establishing causal relationships is challenging. Moreover, as data collection relied on self-reporting, there may be some degree of information bias. Future research could consider employing a longitudinal design to ascertain the causal relationship between HL and smoking behavior, and attempt to use more objective measurement methods, such as blood nicotine levels, to minimize the potential for reporting bias.

## Conclusion

This study underscores the potential value of enhancing HL to reduce smoking behavior. These findings provide strong scientific support for the design and implementation of public health strategies and health education interventions. Notably, the nonlinear relationship between HL and smoking behavior identified in this research offers new insights for crafting more precise and effective strategies for smoking control. Furthermore, considering the influence of education level, occupational type, and social support, these strategies should integrate socio-economic factors to design comprehensive and targeted health promotion plans.

This research demonstrates that (1) residents with higher levels of HL have significantly lower smoking rates compared to those with lower HL, and (2) smokers with higher HL consume fewer cigarettes daily. (3) Additionally, non-smokers with higher HL are more inclined to discourage others from smoking, highlighting the crucial role of HL in preventing and controlling smoking behavior. (4) The study also reveals a nonlinear relationship between HL and smoking behavior through RCS analysis. (5) Moreover, the findings indicate significant differences between smokers and non-smokers across various dimensions, including age, education level, occupational category, marital status, and awareness of health.

## Data Availability

The raw data supporting the conclusions of this article will be made available by the authors, without undue reservation.
